# Closing the Stable Door on Strangles: Serological Responses of Vaccinated Horses on a Farm Following the Arrival of a New Horse †

**DOI:** 10.3390/ani15243584

**Published:** 2025-12-13

**Authors:** Erika Rask, Francesco Righetti, Aymé Ruiz, Joakim Bjerketorp, Sara Frosth, Lars Frykberg, Karin Jacobsson, Bengt Guss, Jan-Ingmar Flock, Birgitta Henriques-Normark, Emma Hartman, Agneta Gustafsson, Romain Paillot, Andrew S. Waller

**Affiliations:** 1 Veterinär Erika Rask AB, 442 32 Kungälv, Sweden; 2 Department of Microbiology, Tumor and Cell Biology, Karolinska Institutet, 171 77 Solna, Sweden; 3 Mybac, 129 22 Stockholm, Sweden; 4 Department of Animal Biosciences, Swedish University of Agricultural Sciences, 750 07 Uppsala, Swedensara.frosth@slu.se (S.F.);; 5 Clinical Microbiology, Karolinska University Hospital Solna, 171 76 Stockholm, Sweden; 6 Intervacc AB, 129 22 Stockholm, Swedenandrew.waller@intervacc.com (A.S.W.)

**Keywords:** DIVA, horse, Strangvac, *Streptococcus equi*

## Abstract

Infection of susceptible horses with *Streptococcus equi* subspecies *equi* (*S. equi*), the causative agent of strangles, is associated with commingling. Exposure may occur among horses at equestrian events, sales, or horses moved among different equine stabling environments. Strangles can affect all horses on a farm, leading to the death of up to 10% of cases depending on their immunity status at the time of infection, the development of complications, the success of biosecurity measures, and the use of vaccination. The current retrospective study used ELISAs to measure the exposure of horses to *S. equi* at a farm that experienced an outbreak of strangles shortly after the introduction of a new horse on the same day that the majority of the 17 resident horses were vaccinated with Strangvac for the first time. One vaccinated horse, which subsequently tested positive for *S. equi* and EHV-4, developed a cough, elevated body temperature, and nasal discharge 11 days after the first vaccination. Two other horses developed fever for one day at 22 days post-first vaccination, but only one had serological evidence of exposure to *S. equi*. All vaccinated horses had high antibody titres to vaccine components, whilst 7 of the 17 resident horses, and the new arrival, tested seropositive for exposure to *S. equi*. Although 3 out of the 17 vaccinated horses developed mild signs of disease before second vaccination, serological data support the effectiveness of vaccination in resident populations of horses to minimise the risk of strangles following the introduction of a new horse.

## Simple Summary

Strangles, caused by *Streptococcus equi* subspecies *equi* (*S. equi*), is one of the most frequently diagnosed infectious diseases of horses worldwide. Serological testing can be utilised to identify horses that were exposed to *S. equi* and thus determine the effectiveness of preventative measures. Here, we report the use of two ELISA assays that differentiate infected from vaccinated animals (DIVA), to determine the exposure of horses to *S. equi* following the introduction of a new horse on the same day that the majority of the 17 resident horses began their vaccination programme against strangles. Three of the resident vaccinated horses experienced mild clinical signs of disease from 11 days after arrival of the new horse, but only one had confirmed *S. equi* infection. All vaccinates responded to immunisation and no new cases of disease occurred following the administration of a second dose of vaccine, whilst serological analysis showed that seven of the resident vaccinated horses, and the new arrival, had been exposed to *S. equi*. Our data support the use of vaccination in resident populations of horses to minimise the risk of strangles prior to, or immediately following, the arrival of a new horse.

## 1. Introduction

Strangles is an infectious disease of the horse caused by the bacterium *Streptococcus equi* subspecies *equi* (*S. equi*). *S. equi* is transmitted via direct contact with an infected horse or contaminated fomites and the infection is endemic in most populations of horses worldwide, with several hundred outbreaks reported every year [1,2,3,4,5]. Strangles is characterised by pyrexia, pharyngitis, and lymph node (LN) abscess formation, most commonly in the head and neck regions. Abscess rupture, particularly from the retropharyngeal LNs, is characterised by mucopurulent nasal discharge. The severity and duration of clinical signs of strangles vary depending on the level of exposure to *S. equi* and the immune status of the horse. Morbidity in susceptible horse populations can be very high, reaching 100% [6]. Severe complications such as metastatic abscessation (also known as bastard strangles), purpura haemorrhagica and immune-related myopathies may occur [7,8], with reported complication rates of up to 20% [9,10,11]. Mortality is variable, but can exceed 10% in some outbreaks where the level of exposure to *S. equi* is high [10,12].

Approximately 10% of horses that recover from strangles remain persistently infected providing an important reservoir of *S. equi* in farms with endemic disease [13,14]. However, long-term persistently infected carriers shed *S. equi* intermittently and the strains may have a reduced level of fitness [15,16]. Recent research found that a rapid change in the population of *S. equi* that was recovered from horses in the UK was inconsistent with the majority of outbreaks being caused by the movement of persistently infected horses [17]. McGlennon et al. proposed that the transmission of *S. equi* from acutely infected/recently convalesced short-term carriers was likely responsible for the majority of new outbreaks in the UK between 2016 and 2022 [17]. Such findings highlight the importance of interventions including quarantine, isolation and biosecurity measures, serological screening, and vaccination towards reducing the impact of strangles.

*S. equi* infection generates a strong humoral immunity [18], including antibody responses to two immunodominant sortase-processed surface proteins, SEQ_2190 (antigen A) and SeM (antigen C). The SEQ_2190 and SeM antigens that are utilised in the A/C iELISA are not included in Strangvac, a recombinant protein-based vaccine against strangles [19]. Therefore, vaccination with Strangvac does not lead to positive test results in the A/C iELISA, or other diagnostic serological, PCR or culture assays and these tests can be utilised to differentiate infected from vaccinated animals, so-called ‘DIVA’ [19]. Strangvac contains fragments of the *S. equi* cell surface proteins CNE, SclC, SclF, SclI, and EAG that are fused together to form the vaccine antigen CCE, fragments of the cell surface proteins Eq8 and Eq5 that are combined as vaccine antigen Eq85, the secreted protein IdeE, and a saponin-based adjuvant (Novavax Inc., Gaithersburg, MA, USA) [19]. Strangvac protected 94% of horses against a high-dose experimental infection with *S. equi* in one of the clinical studies [19] and is available commercially in Europe [4]. However, little is known about the protective effects of Strangvac under field conditions and whether it is possible to use vaccination as a tool to mitigate the risk of strangles in resident horses following the arrival of a new horse.

This retrospective study aimed to provide insights regarding the transmission of *S. equi* following the arrival of a new horse at a farm in Sweden and the protection induced by vaccination. It combined clinical observations and the quantification of antibody levels in response to vaccination and exposure to *S. equi*. Furthermore, a new diagnostic iELISA was developed and evaluated as a more practical alternative for the measurement of antibody responses following the vaccination of horses with Strangvac.

## 2. Materials and Methods

### 2.1. Vaccination, Outbreak Description, and Sampling of Horses

**Figure 1:**
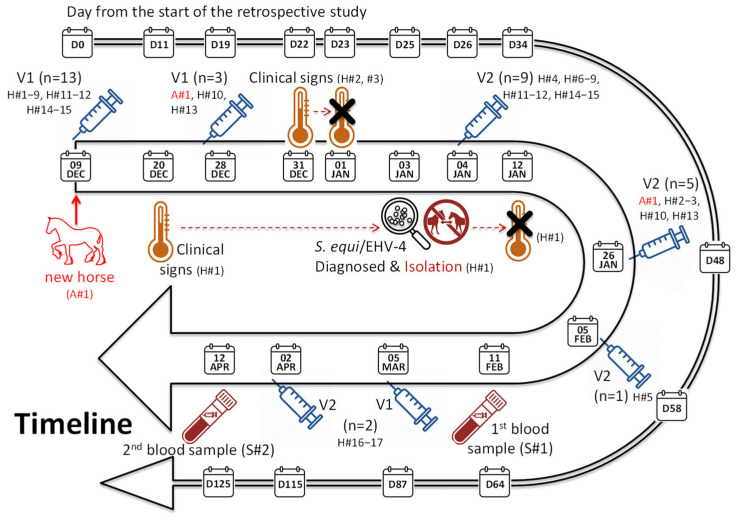
Timeline of events. Vaccination (V1 and V2) and sampling dates are reported. Due to the nature of the resident horses, not all individuals were vaccinated at the same time. n = number of horses concerned with a specific intervention. Clinical signs were observed in three horses. The thermometer icons delineate the first and last (represented with a X) report of clinical signs. The horse ID (H# and A#) is indicated. Both date (day and month) and the number of days from the start of the study (D) are indicated.

This retrospective study reports events that took place at a Swedish equine yard between December 2023 and April 2024. The information reported here were collected as part of the veterinary management and investigative activities in the yard. The yard housed 17 resident competition horses and was also opened to external horses, which visited every week for training. On average, five external horses per week were ridden in an indoor arena at the same time as resident horses. The yard had no reported history of strangles, or other respiratory diseases in the months preceding this study, but the yard owner decided to begin vaccination against strangles due to the frequent movement of horses into, and out of, the yard and the report of several outbreaks nearby. The timeline of the events is summarised in Figure 1 and the Supplementary Table S1. The majority of resident horses (13 out of 17) received their first immunisation (V1) of Strangvac, administered intramuscularly into their neck muscle as per manufacturer’s recommendation, on 09DEC (day 0). On the same day, a new horse (A#1), which was recently purchased from a dealer, entered the herd. Horse A#1 had a veterinary check before arrival, was considered to be in good health, and was not tested or quarantined at the time of arrival. Five of the horses were vaccinated at later dates for the following reasons: A#1 was the new arrival and V1 was administered on 20DEC (day 11), H#10 and H#13 were off-site on the 09DEC and were vaccinated when they returned to the facility (V1 on 20DEC, day 11). H#16 suffered from Pituitary Pars Intermedia Dysfunction (PPID; Cushing’s disease) and H#17 was 26 years of age. Therefore, vaccination of H#16 and H#17 was delayed until 05MAR (V1) and 02APR (V2) due to concerns related to their pre-existing pathology and advanced age, and until the frequency of adverse events after primo-vaccination (V1 and V2) was assessed in the first group of vaccinated horses. The 2nd immunisations were administered to healthy horses between the 04JAN and 05FEB (days 26 and 58, respectively; 31.2 ± 9.6 days after V1).

Three horses (H#1, H#2, and H#3) developed clinical signs of disease. H#1 was a resident horse and did not leave the yard in the month preceding the onset of clinical signs. H#1 developed a mild increase in rectal temperature (38.4 °C) and mild intermittent cough 11 days after V1. Nasal discharge was observed on 31DEC (day 22), which became more profuse on 02JAN (day 24). The presence of both *S. equi* and equine herpesvirus type 4 (EHV-4) was confirmed by qPCR of a sample of nasal discharge taken on day 24 (SVA: Swedish Veterinary Agency, Uppsala, Sweden; results provided on day 25). H#1 was isolated in an outside barn from 03JAN (day 25) for a minimum of 2 weeks (beyond recovery on 12JAN/day 34) and did not receive a second immunisation until 14MAY (day 157; 4 months after clinical recovery). Two further horses (H#2 and H#3) showed mild body temperature increase on 31DEC (day 22) for one day only. H#3 tested negative for *S. equi* and EHV-4 on day 24, and H#2 was not sampled. No further clinical signs associated with *S. equi* infection were observed.

Blood sera were collected from all horses 2 months apart (61 days) as part of veterinary health checks on 11FEB and 12APR (S#1 and S#2; 64 and 125 days after the arrival of A#1, respectively). Sera were quantified for total antibody titres to vaccine antigens CCE, Eq85 and/or IdeE [19], and to antigen A and antigen C in the A/C iELISA, which is unaffected by the immune response post-vaccination with Strangvac and hence is DIVA-compatible [20].

### 2.2. CCE, Eq85, and IdeE Antigen iELISAs

Total antibody titres to CCE, Eq85, and/or IdeE were measured by iELISA using a modified version of the method previously described [21]. Briefly, 96-well plates (ThermoFisher, Waltham, MA, USA) were coated overnight with 100 µL of CCE, Eq85, and IdeE (Swedish University of Agricultural Sciences, Uppsala, Sweden), which were mixed together at a concentration of 4 µg per ml of each antigen in PBS. Plates were then blocked with 2% bovine serum albumin in PBS for 1 h at 37 °C. Plates were washed with PBS-0.05% Tween 20 (PBST) and a two-fold dilution series of equine serum samples in PBST from 1:20 to 1:40,960 were added to triplicate wells. The plates were incubated for 2 h at 37 °C, washed with PBST, and 100 µL of a 1:10,000 dilution of anti-horse-IgG antibody conjugated with horseradish peroxidase (Sigma, Burlington, MA, USA) was added. The plate was incubated for a further 1 h at 37 °C and washed with PBST. A total of 100 µL of TMB substrate (ThermoFisher, Waltham, MA, USA) was then added to each well, the plate was incubated for 10 min, and the reaction was stopped by addition of 100 µL of 1M HCl. The OD_450nm_ was measured and the log_10_ value of the dilution required to obtain an absorbance value below a cut-off threshold of 1.5 was calculated (i.e., Strangvac Ab titre). A positive result was indicated by an antibody titre of ≥3.0.

A modified version of the CCE, Eq85, and/or IdeE iELISA was also developed and performed as above with the exception that only one dilution (1:10,000) of equine serum samples was tested. Results are expressed as the OD_450nm_ measurement. Negative and positive sera were used as controls in both assays (archived sera from the study published by Robinson et al. (2020) [19]). A positive result was indicated by an OD_450nm_ of ≥0.5.

### 2.3. Dual Antigen A/C iELISA

A total of 100 µL of equine serum was diluted 1:800 in PBST and 1% non-fat milk and analysed as described previously [20]. Assays were performed in duplicate and OD_450nm_ data were normalised using control sera run on each assay plate. The normalised OD_450nm_ data from the duplicate assays were averaged and an OD_450nm_ of ≥0.5 was considered to be a positive result for antigen A or antigen C iELISAs [20].

### 2.4. Statistical Methods

Normality was tested with the Shapiro–Wilk test. Data were found to be normally distributed. The paired Student’s *t*-test was used to compare vaccine antigens titres measured at S#1 and S#2. The Pearson’s correlation test was used to determine if there was a significant relationship between the results from the classical and modified CCE, Eq85, and IdeE antigen iELISAs [22], with significance set at *p* < 0.05 (Statistics Kingdom 2017. Available from: http://www.statskingdom.com).

## 3. Results

### 3.1. Adverse Events After Immunisation

A total of 36 doses of Strangvac were administered. A transient elevation of rectal body temperature and/or local swelling was recorded on four occasions each (11.1%). A transient depression was recorded on 12 occasions (33.3%). These transient reactions were observed 24 to 48 hr after immunisation and lasted one to four days. Both horses H#16 (PPID) and H#17 (26 years old) showed no reaction to vaccination.

### 3.2. Serological Response to Vaccination

**Figure 2:**
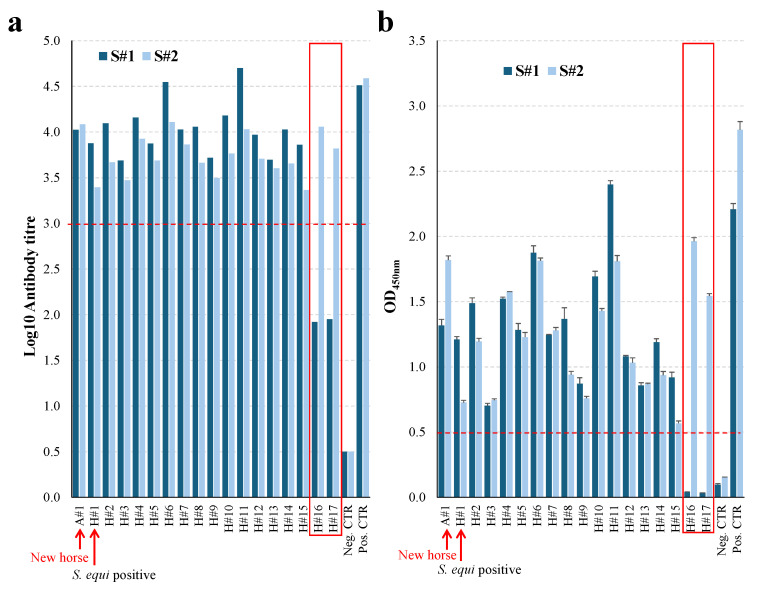
Serological response to vaccination. Serum samples collected at S#1 and S#2 were analysed with the conventional or single dilution CCE, Eq85, and/or IdeE iELISAs (**a**,**b**), respectively). Results are expressed as log_10_ antibody titre (**a**) or average mean OD_450nm_ ± STDV (**b**). For the conventional iELISA (**a**), the cut-off for positivity was ≥3.0 and for the single dilution iELISA, (**b**) the positive cut-off was an OD_450nm_ of ≥0.5 (red dotted lines). Horses H#16 (PPID) and H#17 (26 years old), which were not vaccinated at the time of S#1, are boxed. The *S. equi*-positive horse (H#1) and the new horse (A#1) are indicated. The negative and positive controls were used with both set of samples (S#1 and S#2).

Only two horses (H#16 and H#17) were unvaccinated at the time of the first blood sample (S#1) at 64 days after the arrival of A#1. They both had a negative titre at S#1 (titre < 3.0), and a low level of anti-CCE, Eq85, and/or IdeE antibodies measured in the single dilution iELISA (OD_450nm_ < 0.5). H#16 and H#17 seroconverted and tested positive in both of these iELISAs (titre ≥ 3.0 and OD_450nm_ ≥ 0.5) when these horses were sampled two months later (S#2; 38 days after V1 and 10 days after V2). Blood samples were collected from H#1 at 64 (S#1) and 125 (S#2) days after V1, which was 53 and 114 days after the onset of clinical signs of strangles, and 39 and 100 days after the confirmation of *S. equi* infection, respectively. Horse H#1 was seropositive for antibodies to CCE, Eq85, and/or IdeE at both S#1 and S#2 in both iELISAs. For all other horses, S#1 was taken between 6 and 38 days after V2 and all samples (S#1 and S#2) were positive in both iELISAs. Excluding H#16 and H#17, the average antibody titre significantly decreased between S#1 and S#2 (4.03 ± 0.28 and 3.72 ± 0.23, respectively; *p* < 0.001). The individual serological antibody titres to CCE, Eq85, and/or IdeE are presented in Figure 2a and Supplementary Table S1, whilst the level of antibodies to CCE, Eq85, and/or IdeE measured in the single dilution (1:10,000) assay are presented in Figure 2b and Supplementary Table S1.

### 3.3. Serological Response to S. equi Exposure

**Figure 3:**
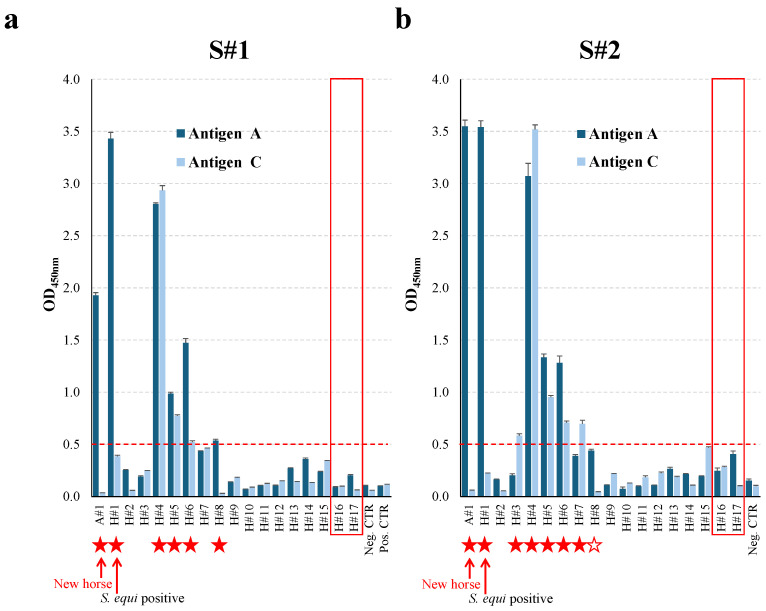
Serological response to *S. equi* exposure/infection. Serum samples collected at S#1 (**a**) and S#2 (**b**) were analysed with the dual antigen A/C iELISA. Results are expressed as average meant OD450 nm ± STDV, with a cut-off for positivity set up at ≥0.5 OD450 nm (red dotted line). Results with both antigens A and B are represented. Positive horses (positive for at least one antigen) are indicated with a red star. The open star indicates a horse positive for exposure at S#1 but negative at S#2. The *S. equi*-positive horse (H#1) and the new horse (A#1) are indicated. Horses H#16 and H#17, which were not vaccinated at the time of S#1, are boxed.

Six (A#1, H#1, H#4, H#5, H#6, and H#8) out of 18 horses tested seropositive for exposure to *S. equi* when sampled at S#1, which was 64 days post-arrival of A#1 and 53 days after the start of clinical signs in H#1. Sample S#2, which was collected 61 days later, remained seropositive for horses A#1, H#1, H#4, H#5, and H#6. Antibody levels in horse H#8 returned to a seronegative level at S#2, but horses H#3 and H#7 tested seropositive. Overall, combining the results at S#1 and S#2, 8 horses out of 18 (44.4%) showed serological evidence of exposure to *S. equi*, including H#1 with confirmed *S. equi* infection and A#1, which was the new horse. The individual serological response to *S. equi* infection or exposure is presented in Figure 3 and Supplementary Table S1.

**Figure 4:**
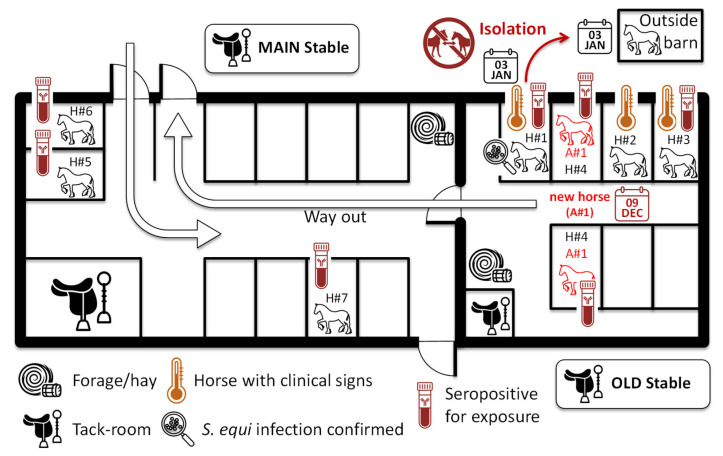
Map of the stable block and horse location. When known, the location of horses is indicated, with an illustration of their serological status for exposure to *S. equi* and clinical signs. Horses A#1 and H#4 exchanged stable box around one month after the arrival of A#1.

A map of the stable block is presented in Figure 4. Horse A#1 arrived at the farm on day 0 and, having passed a veterinary check, was allowed to enter the stable block without testing or the introduction of quarantine measures. Horses H#1, A#1, and H#4, which had the highest *S. equi* antibody levels at both S#1 and S#2, were housed in adjacent stable boxes. H#1 began showing clinical signs of strangles on day 11, but infection with *S. equi* was not diagnosed until day 25 and this horse was isolated the same day for a minimum of 2 weeks. Horses A#1 and H#4 exchanged stable boxes in January, around 1 month after the arrival of A#1, two weeks after the start of clinical signs in H#1 and shortly after H#4 had received a second dose of vaccine.

Horses H#2 (seronegative for exposure to *S. equi*) and H#3 (seropositive for exposure to *S. equi*) were kept in stable boxes adjacent to H#1, A#1, and H#4 when being groomed a few times each week, but were otherwise kept outdoors. The stable boxes adjacent to H#4 were not used by other horses. Horses H#5 and H#6, which were also seropositive for exposure to *S. equi*, were housed near the stable entrance/exit and the main tack room. Horse H#7 was kept in one of the stable boxes on the main row. The location of other horses was not recorded. The attending veterinarian noted that five out of the eight horses that tested seropositive for exposure to *S. equi* (A#1, H#4, H#5, H#6, and H#7) were worked together on a daily basis until *S. equi* was diagnosed (day 25). H#4 shared a paddock with H#1 when these horses were turned out.

### 3.4. Comparison of the Conventional and Single Dilution Vaccine Antigen iELISA

**Figure 5:**
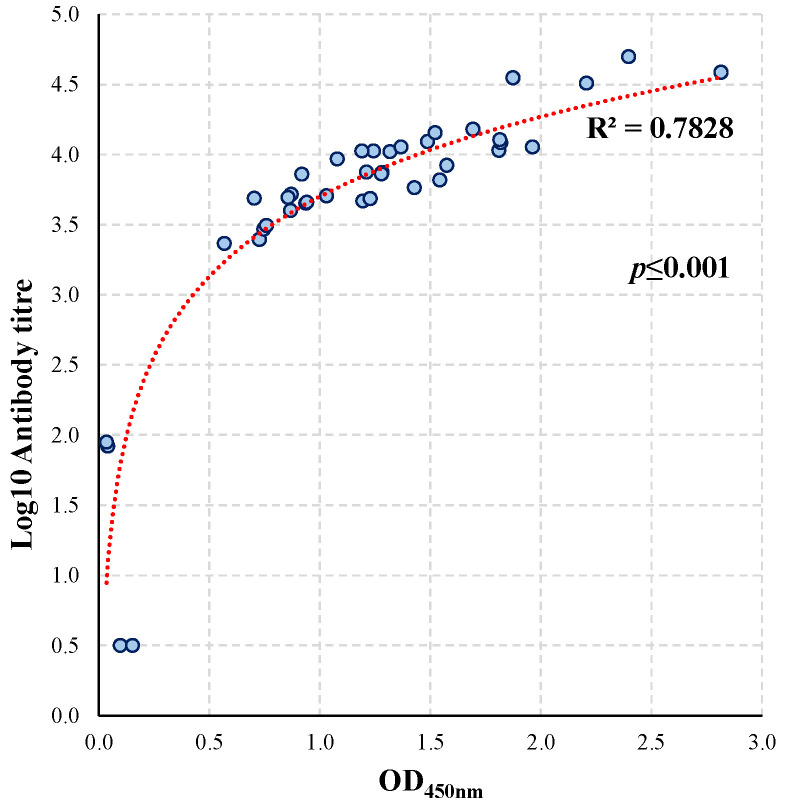
Comparison of the conventional and single dilution CCE, Eq85, and/or IdeE antigen iELISAs. Correlation analysis with all serum samples considered (n = 40; S#1 and S#2), including controls. The R^2^ and logarithmic regression curves are presented. Significance was set at *p* ≤ 0.05 (Pearson’s correlation test).

Results from the vaccine antigen iELISAs (conventional and single dilution) were compared (Figure 5). The Pearson correlation test indicated that there was a significant strong positive relationship between the vaccine antigen antibody titres obtained with the classical iELISA and the OD_450nm_ measurement in the single dilution iELISA (r(38) = 0.793, *p* < 0.001) [22].

## 4. Discussion

The main objectives of this retrospective study were to understand how *S. equi* was introduced to this yard and to determine the level of exposure of recently vaccinated horses to *S. equi*.

### 4.1. Possible Route of Introduction of S. equi

Retrospective epidemiological studies are observational by nature and present certain limitations as samples collected prior to a clinical event are often lacking and clinical data may be incomplete. It was not possible to confirm the serological status of this herd prior to the introduction of the new horse (A#1) as the first blood sample was taken 64 days afterwards. However, no cases of strangles were reported in this population of horses prior to the arrival of A#1. Ten of the 17 resident horses tested seronegative for exposure to *S. equi* in the antigen A/C iELISA when sampled at S#1 or S#2, indicating that the majority of resident horses were either not exposed to *S. equi* prior to, or during the outbreak, or that they were exposed to a sufficiently low dose that an immune response to antigens A or C was not stimulated. In particular, horses H#16 and H#17, which were not vaccinated until 05MAR due to pre-existing PPID and old-age, respectively, were kept separate from A#1 and tested seronegative for exposure to *S. equi* in the antigen A/C iELISA. Therefore, as antibody responses induced by exposure to *S. equi* persist for several months [23,24], it is likely that *S. equi* was not circulating at a subclinical level in this herd in the weeks before the arrival of A#1.

A#1 was in good health immediately before arrival and remained healthy throughout the subsequent outbreak. A#1 had high levels of antibodies to antigen A at both S#1 and S#2, but no pre-arrival serological and/or bacteriological testing was conducted. Therefore, it is not possible to confirm if A#1 had been exposed to *S. equi* prior to arrival at the farm and if indeed this horse was the source of the outbreak. However, given the probable lack of *S. equi* circulation at the farm prior to the arrival of A#1, it is likely that this horse was the source of infection either from having recently recovered from strangles, or through being a subclinical carrier of *S. equi*. It is recommended to quarantine and screen new horses for 2 to 4 weeks, to provide time for those horses incubating an infectious disease to begin showing clinical signs, and to provide an opportunity to identify and treat subclinical persistently infected horses [9,25]. However, this biosecurity measure is not always possible, practicable, or implemented, which increases the risk of the introduction of pathogens. A#1 was not screened for the presence of infectious agents or isolated on arrival and so there were no biosecurity measures in place at the farm to mitigate the risk of transmission to resident horses. It is believed that A#1 had not been vaccinated against strangles before arrival and, therefore, the first vaccine dose was administered to A#1 on day 19.

**Key message:** As noted by Lakic et al., (2024), the majority of strangles outbreaks are linked to the introduction of a new horse into the population [26]. Inadequate, or absent, biosecurity protocols and diagnostic testing substantially increase the risk of *S. equi* transmission and subsequent dissemination within the herd.

### 4.2. Transmission of S. equi in the Yard

The results of the antigen A/C iELISA provide insights into the transmission of *S. equi* during this strangles outbreak. Aside from A#1, horses H#1 and H#4 had very high antibody levels when measured at S#1 and S#2. Horse A#1 arrived at the time of first vaccination (V1) of 13 of the 17 resident horses on day 0. A#1 was housed next to H#1, which developed clinical signs of strangles from day 11 and was diagnosed with *S. equi* infection on day 25 (sampled on day 24). The start of clinical signs of disease in H#1, at 11 days post-arrival of A#1, is consistent with the expected incubation period of *S. equi* infection and the typical time to onset of disease [9]. The very high antibody levels to antigens A and C at S#1 and S#2 in H#4 also indicate exposure to *S. equi*. However, it is not possible to determine if H#4 was exposed to *S. equi* via contact with A#1 shortly after the arrival of this horse, as was likely for H#1, or if the exposure of H#4 occurred somewhat later, either after contact with *S. equi* shed from H#1, or in January following movement to the stable box that had been occupied previously by A#1. The survival of *S. equi* in the environment is generally poor, but it can persist for several days in the absence of direct sunlight and/or in wetter conditions [9,27,28]. It is not known if the stable boxes were disinfected before the A#1/H#4 swap. A later date of exposure of H#4 to *S. equi* could have been sufficient for the development of immunity in this horse following a second vaccination on day 26, which provides one explanation for the lack of clinical signs despite the extremely high antibody levels to antigens A and C that were more typical of horses with acute strangles.

While horse H#1 displayed clinical signs of strangles for three weeks, only two other horses (H#2 and H#3) developed transient mild clinical signs of fever for one day at 22 days after first vaccination. Both H#2 and H#3 were in proximity to H#1, A#1, and H#4 when being groomed in adjacent stable boxes. H#3 was found to be seropositive to *S. equi*, but only when sampled 103 days (S#2) after having an elevated temperature. H#2 tested seronegative at both S#1 and S#2. Four other horses showed serological evidence of exposure to *S. equi* (H#5, H#6, H#7, and H#8). While these horses were housed in different parts of the stable, H#5, H#6, and H#7 worked together with A#1 and H#4 daily, which could have provided an opportunity for direct exposure to *S. equi*. Their antigen A/C antibody levels were lower than in H#1, A#1, and H#4, which may indicate a lower level of exposure to *S. equi*.

An alternative explanation for this outbreak is the introduction of *S. equi* from one of the horses visiting the farm for training purposes, which occurred on a weekly basis. In this scenario, a visiting horse may have transmitted *S. equi* to H#1, which may then have exposed the other horses. H#1 was isolated in an outside barn when diagnosed with *S. equi* infection, 15 days after the onset of disease. *S. equi* shedding may occur as early as one or two days after the onset of pyrexia [9], which provides a potential window of transmission to A#1 and H#4. In this scenario, the arrival of A#1 was coincidental.

**Key message:** Integration of epidemiological data with serological analysis provides a clearer understanding of the introduction and transmission pathways of *S. equi*, and the evidence strongly suggests that horse A#1 was the primary source of the strangles outbreak, with H#1 exhibiting clinical signs consistent with direct exposure.

### 4.3. Impact of Strangles Vaccination

The absence of an unvaccinated control group limits the analysis of the role played by vaccination during this outbreak. Two horses (H#16 and H#17) remained unvaccinated for three months from the start of the outbreak and can be considered as sentinel controls. H#16 and H#17 tested seronegative in the antigen A/C iELISA at S#1 and S#2 and so there was no evidence that they had been exposed to *S. equi* during the outbreak. H#16 and H#17 also tested negative at S#1 for antibodies to the vaccine components, which confirmed their lack of humoral immunity to *S. equi* prior to V1. Both of these horses tested positive for antibodies to the vaccine components at S#2, which was 10 days after the second vaccination. It is possible that herd immunity to *S. equi* that was induced in the other horses by V1 and V2, in combination with the biosecurity measures that were put in place following the confirmation of *S. equi* infection in H#1 on day 25, prevented transmission of *S. equi* to H#16 and H#17, and the other non-exposed horses, which may have minimised the severity and duration of this outbreak.

Potentially, the onset of protection against *S. equi* circulation was rapid, as the first vaccination took place only 11 days before the onset of clinical disease in H#1. H#1 was not isolated for another 15 days, and A#1 was not isolated during the outbreak. A rapid onset of protection induced by Strangvac was suggested recently by Gröndahl et al. (2025), when this vaccine was used to control another outbreak of strangles in Sweden following the identification of three non-vaccinated clinical cases [29]. The remaining seventeen healthy horses were vaccinated 23 days after the onset of clinical signs. None of the vaccinates developed signs of strangles despite nine of the 17 horses (59%) testing seropositive in the antigen A/C iELISA, indicating exposure to *S. equi*. All three unvaccinated clinical cases were seropositive in the antigen A/C iELISA and two of the three cases had to be euthanised after developing severe clinical signs [29].

Strangvac induces significant levels of antibodies against each of the vaccine components from 8 days post V1 in naïve ponies [19]. This could be the consequence of the vaccine adjuvant used and/or the prior exposure of vaccinated animals to *Streptococcus equi* subspecies *zooepidemicus* (*S. zooepidemicus*). A recent study by Reemers et al. (2020), measured detectable equine influenza-specific antibody titres 9 days after the first administration of an equine influenza (EI) ISCOMatrix-adjuvanted vaccine, which utilises a similar adjuvant to Strangvac [30]. However, this result was unusual as longer seroconversion times were reported previously for the same EI vaccine [31]. Immunological priming linked to prior exposure to *S. zooepidemicus* provides another explanation for the rapid immune response to Strangvac. The opportunistic pathogen *S. zooepidemicus* shares over 97% genome identity with *S. equi* [32], and it is endemic in horse populations and isolates of *S. zooepidemicus* encode at least four Strangvac antigens with 80.2% to 99.3% amino acid sequence identity (Frosth S., manuscript in preparation).

Exposure to, and infection with, *S. equi* is usually associated with high morbidity in susceptible horse populations, but defining an accurate morbidity rate is complex as it is dependent on the specific biosecurity measures that are implemented to minimise the exposure of horses to *S. equi*, and the immune status of horses at the time of exposure. All 41 unvaccinated Icelandic horses at a farm in Sweden tested positive in the A/C iELISA, indicating exposure to *S. equi*, and all of these horses developed clinical signs of strangles [24]. A morbidity rate of 53% was reported by Tschelschlok et al. (2018) [33] during a strangles outbreak in a group of 112 unvaccinated weanlings, of which 91 (81%) tested seropositive in the antigen A/C iELISA [33]. However, the circulating strain in this outbreak contained a 61 bp deletion in the SEQ_0402 gene, which encodes the Eq8 antigen used in Strangvac, and the authors speculated that this may have reduced its virulence [33]. Boyle et al. (2017) reported a median duration of clinical signs of 10 days (interquartile range, 7 to 21 days) [23]. Christmann and Pink reported an average duration of 14 days (range from 1 to 84 days, depending of the treatment that was administered) [10]. Severe complications were also reported in these studies. Results from the Surveillance of Equine Strangles (SES) website over a period of 10 years (05JAN2015 to 03DEC2025) indicated that of the 3494 reported diagnoses of *S. equi* infection, 1495 (42.8%) horses were reported to have clinical signs of disease on the diagnostic submission form [5]. Based on the UK data set, clinical cases in field outbreaks were characterised as having nasal discharge (71%), fever (47%), LN swelling (29%), coughing (24%) and LN abscessation (17%), either alone or in combination [5,10,23]. Strangvac was launched in the UK during AUG2022, but none of the 1059 diagnosed cases of *S. equi* infection since this time (up to 03DEC2025) were reported to have been vaccinated with Strangvac.

In the current study, H#1 displayed mild clinical signs of strangles for 24 days and H#2 and H#3 had fever for one day in the period between first and second vaccination. However, the raised body temperature in H#2 and H#3 may not have been related to *S. equi* as H#2 did not test positive in the antigen A/C iELISA and, whilst H#3 tested seropositive at S#2, and this horse was seronegative at S#1. When sampled on day 24, H#3 did not test positive for the presence of *S. equi* (H#2 was not sampled or tested for the presence of *S. equi*). Therefore, the frequency and severity of disease in this vaccinated population was relatively low (16.7%, 3 out of 18) when compared with the morbidity rate reported in other studies [6,33] and none of the vaccinated horses developed clinical signs of strangles after the second dose of vaccine.

The adverse events observed during the study were transient and similar to reactions previously described [4]. The vaccine used in this study contains only recombinant proteins and a saponin-based adjuvant and therefore presents no risk of inducing strangles. This is in contrast with strangles vaccines that contain live attenuated vaccine strains. Kelly et al. (2006) investigated the diagnoses of *S. equi* infection at 24 yards in the UK between NOV2003 and MAY2005 and found that horses at three yards (13%) had clinical signs from which an attenuated live strangles vaccine strain was recovered [34]. Two cases of strangles were attributed to the use of a live attenuated vaccine in New Zealand [35,36] and one US *S. equi* outbreak isolate was also related to a live attenuated vaccine strain [36].

**Key message:** Although the absence of a true unvaccinated control group limits interpretation, the data suggest that rapid onset of immunity following vaccination, combined with biosecurity measures, likely reduced transmission and minimised the severity and duration of the strangles outbreak.

### 4.4. Co-Circulation of S. equi and EHV-4

Horse H#1 was positive for both *S. equi* and EHV-4 when screened on day 24. EHV-4 is one of the most prevalent respiratory viruses of horses and is considered to be endemic in most horse populations [3]. EHV-4 infection induces mild respiratory clinical signs and long-lasting latency with occasional viral reactivation [37]. A recent bio-surveillance study by Jaramillo-Morales et al. (2023) reported co-detection by PCR of EHV-4 in 8.1% of *S. equi* qPCR positive horses (58 out of 715 cases) [38]. It is not possible to determine if EHV-4 contributed to the clinical signs of disease in H#1 (or H#2 and H#3), if EHV-4 was concomitantly circulating in the herd at the time of *S. equi* infection, or if the presence of EHV-4 in H#1 was due to reactivation of a latent infection [39]. When sampled on day 24, H#3 did not test positive for the presence of EHV-4. The relationship between infection with equine herpes viruses and *S. equi* (and/or *S. zooepidemicus*), and the impact of co-infection on the severity of clinical signs is worthy of further research.

Since the resolution of the outbreak reported in this study, strangles vaccination is mandatory and the herd has received another three booster immunisations with Strangvac (around three months after V2, seven months after V3 and nine months after V4). New arrivals are kept separated for 2 weeks. No further cases of strangles have occurred on the farm. Horses A#1 and H#4 moved to a new stable, which has also not reported any cases of strangles.

### 4.5. Comparison of the Vaccine Antigens iELISAs

Although the iELISA assays used in this study combined the vaccine antigens, Robinson et al. (2020) have demonstrated that immunisation with Strangvac induces an antibody response to individual antigens (i.e., CCE, Eq85, and IdeE) [19]. The functional activity of the antibody response to IdeE has also been recently characterised, showing its ability to neutralise the immunoglobulin-cleaving activity of IdeE in vitro [40]. The measurement of total antibody titres towards the vaccine antigens may, in time, prove to be a useful indicator of the development of protective immunity. However, the measurement of antibody titres is both expensive and time-consuming. Therefore, the antibody titres to the components of Strangvac were compared with antibody levels measured by iELISA using a single 1:10,000 dilution of blood sera, which is both cheaper and quicker to perform. There was a highly significant correlation between the two methodologies and we propose that the single dilution iELISA is an effective assay with which to measure the immune responses to Strangvac vaccination. The wider application of this assay, and the antigen A/C ELISA, in vaccinated populations of horses as they come into contact with *S. equi*, will be useful tools with which to monitor the effectiveness of vaccination for the prevention of strangles in horse populations across the world.

### 4.6. Study Limitations

Given the observational nature of this retrospective study, several inherent limitations must be acknowledged as follows: limited statistical power due to the study being conducted on a single farm; concurrent infection of horse H#1 with both *S. equi* and EHV-4; and the absence of serological testing prior to the introduction of new horses. Furthermore, retrospective investigations often face challenges in obtaining complete and accurate epidemiological records, which can restrict the depth and reliability of the analysis.

## 5. Conclusions

The results reported here demonstrate the importance of isolation and screening of new arrivals in a herd to prevent the introduction of *S. equi*. The use of the antigen A/C iELISA to discriminate infected from vaccinated horses allowed the identification of vaccinated horses that had been exposed to *S. equi*. Such information is important to direct contingency plans for outbreak prevention and management. A combination of strangles vaccination and biosecurity measures was effective to mitigate the consequence of *S. equi* introduction and limit the transmission and clinical impact of strangles in this recently vaccinated herd following the arrival of a new horse.

## Data Availability

The data used in this study are included in the article and Supplementary Table S1. Further enquiries can be directed to the corresponding author.

## Supplementary Materials

The following supporting information can be downloaded at https://www.mdpi.com/article/10.3390/ani15243584/s1, Table S1: Vaccination, clinical signs and serology.

## Abbreviations

The following abbreviations are used in this manuscript:

DIVA	Differentiation of Infected from Vaccinated Animals
EHV-4	Equine HerpesVirus type 4
EI	Equine Influenza
PPID	Pituitary Pars Intermedia Dysfunction
*S. equi*	*Streptococcus equi* subspecies *equi*
*S. zooepidemicus*	*Streptococcus equi* subspecies *zooepidemicus*
