# Supplementary material for: Closing the Stable Door on Strangles: Serological Responses of Vaccinated Horses on a Farm Following the Arrival of a New Horse
Source: Animals (Basel). 2025 Dec 13;15(24):3584. doi: 10.3390/ani15243584 (PMC12729937; doi:10.3390/ani15243584)
Supplement: Supplementary file 1 [file animals-15-03584-s001.zip › animals-3999213-supplementary.pdf]

Title : Closing the stable door on strangles: serological responses of vaccinated horses in a farm following the arrival of a new horse. Rask *et al.*, 2025.

Supplementary Table S1: Vaccination, clinical signs and serology. Dates of vaccination, dates of clinical signs if observed, exposure to *S. equi* (serology, OD_{450nm}, positive ≥0.5) and vaccine antigens serology (Log10 Antibody titre, positive ≥3). Positive results are reported in blue. ns = not sampled.

Horse ID	V1 (date)	V2 (date)	Clinical signs	*S. equi* testing (PCR)	*S. equi* exposure Antigen A	*S. equi* exposure Antigen C	*S. equi* exposure Antigen A	*S. equi* exposure Antigen C	Vaccination (serology)	
					S#1 (Day 64)		S#2 (Day 125)		S#1 (Day 64)	S#2 (Day 125)
A#1	Day 19	Day 48	none	ns	1.93	0.04	3.55	0.06	4.02	4.09
H#1	Day 0	Day 157	Day 11 – 33	Positive	3.43	0.39	3.54	0.22	3.88	3.40
H#2	Day 0	Day 48	Day 22 only	ns	0.25	0.06	0.16	0.05	4.09	3.67
H#3	Day 0	Day 48	Day 22 only	Negative	0.19	0.25	0.20	0.58	3.69	3.47
H#4	Day 0	Day 26	none	ns	2.80	2.94	3.07	3.52	4.16	3.93
H#5	Day 0	Day 58	none	ns	0.99	0.77	1.33	0.95	3.87	3.69
H#6	Day 0	Day 26	none	ns	1.47	0.52	1.28	0.71	4.55	4.11
H#7	Day 0	Day 26	none	ns	0.44	0.46	0.39	0.70	4.03	3.86
H#8	Day 0	Day 26	none	ns	0.54	0.03	0.44	0.04	4.06	3.66
H#9	Day 0	Day 26	none	ns	0.14	0.18	0.11	0.22	3.72	3.49
H#10	Day 19	Day 48	none	ns	0.07	0.09	0.07	0.13	4.18	3.76
H#11	Day 0	Day 26	none	ns	0.11	0.12	0.10	0.19	4.70	4.03
H#12	Day 0	Day 26	none	ns	0.11	0.15	0.11	0.22	3.97	3.71
H#13	Day 19	Day 48	none	ns	0.27	0.14	0.27	0.19	3.69	3.60
H#14	Day 0	Day 26	none	ns	0.36	0.13	0.21	0.11	4.03	3.65
H#15	Day 0	Day 26	none	ns	0.24	0.34	0.19	0.47	3.86	3.37
H#16	Day 87	Day 115	none	ns	0.09	0.10	0.25	0.28	1.92	4.06
H#17	Day 87	Day 115	none	ns	0.20	0.06	0.41	0.10	1.95	3.82
